# International advocacy for ketogenic diet research in schizophrenia: insights from a public petition

**DOI:** 10.3389/fpubh.2026.1854868

**Published:** 2026-07-14

**Authors:** Sidney L. Murray, Daniel J. O. Roche, Christopher M. Palmer, Shuo Chen, Hwiyoung Lee, Gopal Vyas, Heather A. Adams, Valerie Harrington, Deanna L. Kelly

**Affiliations:** 1 Maryland Psychiatric Research Center, Catonsville, MD, United States; 2 University of Maryland Baltimore School of Medicine, Baltimore, MD, United States; 3 Harvard Medical School, Boston, MA, United States; 4 Spring Grove Hospital Center, Catonsville, MD, United States

**Keywords:** dietary interventions, ketogenic diet, mental health, petition, research advocacy, schizophrenia

## Abstract

Schizophrenia is associated with substantial disability, persistent symptoms, and significant unmet treatment needs. The ketogenic diet has emerged as a metabolic intervention of growing interest for serious mental illness. This study reports quantitative and qualitative findings from a large international Change.org petition advocating for the resumption of a halted randomized blinded inpatient study investigating a ketogenic diet intervention for individuals with schizophrenia. The petition received 21,637 signatures and 1,105 comments from individuals across 151 countries. Participant comments were analyzed using top-down theme consensus coding and Latent Dirichlet Allocation (LDA) topic modeling. Results revealed widespread interest in nutritional and metabolic approaches to mental health, concerns regarding the limitations of current treatment options, and support for further investigation of ketogenic interventions in schizophrenia. Many petitioners described personal or observed experiences with ketogenic diets and emphasized the need for additional research. As this was a self-selected advocacy-driven sample, findings may overrepresent individuals supportive of ketogenic diet research and are not representative of the broader population. These findings provide insight into the perspectives and experiences of petitioners and highlight interest in expanding research on novel treatment approaches for schizophrenia, such as ketogenic dietary interventions.

## Introduction

Schizophrenia is a serious mental illness that consists of positive (e.g., hallucinations and delusions), negative (e.g., blunted affect and anhedonia), and cognitive symptoms (e.g., slow processing speed), with the latter two categories being associated with functional disability (1). Antipsychotic medications are often just partially effective for positive symptoms and are even less effective for negative and cognitive symptoms (1). Antipsychotics also have serious side effects, which include metabolic abnormalities (2). Therefore, novel therapeutic advances that target mechanistic underpinnings of schizophrenia are needed.

Dietary interventions are increasingly being investigated as potential adjunctive treatments for neuropsychiatric disorders. Among these, the medical ketogenic diet has attracted growing interest because of emerging clinical evidence that suggests potential benefits across a range of psychiatric conditions, including schizophrenia (3–7). The medical ketogenic diet is a high-fat, moderate-protein, and low-carbohydrate dietary intervention that induces ketosis, a metabolic state in which ketone bodies become a primary energy source in place of glucose. Interest in the ketogenic diet for schizophrenia is partly motivated by evidence of metabolic abnormalities associated with the disorder, including alterations in glucose metabolism and mitochondrial dysfunction (8). Although the mechanisms underlying potential clinical benefits of the ketogenic diet remain incompletely understood, several pathways have been proposed, including effects on cellular energy metabolism, inflammation, oxidative stress, glutamate signaling, and insulin sensitivity (8, 9).

As randomized, well-controlled clinical trials are needed to establish efficacy for this emerging treatment, our research group began a 5-week randomized single-blind clinical trial testing the efficacy of the ketogenic diet for schizophrenia treatment. This trial took place at our long-standing joint inpatient psychiatric hospital/academic research program (NCT05968638). In this highly regulated setting, participants were randomized to either a ketogenic diet or a standard diet in addition to their existing treatments. Participants assigned to the ketogenic diet received a dietary intervention with a 2.5:1 ratio of fat to combined protein and carbohydrates. To provide all participants with the opportunity to receive the intervention, those initially assigned to the standard diet were offered the ketogenic diet after completion of the 5-week study period. Participants were required to be recommended by their treating psychiatrist, meet predefined eligibility criteria, and undergo assessment of medical decision-making by two independent evaluators. Continuous monitoring and additional safety measures were maintained throughout the study. To our knowledge, this was the first randomized inpatient clinical trial of the ketogenic diet for individuals with serious mental illness conducted in the U. S. This study was approved by the University of Maryland Institutional Review Board (IRB), the Spring Grove Hospital Research Committee, the Maryland Department of Health IRB through a reliance agreement, and an independent Data Safety Monitoring Board. The protocol was also approved under 45 CFR Part 46, SubPart C of the Common Rule, which also provides additional protections for the inclusion of individuals who are court-ordered.

In the Spring of 2024, a system-wide directive within the Maryland state inpatient hospital system discontinued non-federally funded research activities, resulting in the suspension of this ongoing clinical trial, which was supported by private and philanthropic funding sources independent of advocacy (listed in disclosures). The trial was discontinued as a consequence of the directive and not in response to any participant safety concerns. Following study discontinuation, a psychiatrist independent of the study team initiated an international Change.org petition to begin advocacy efforts and bring broader attention to this study halting.^1^ Based on his expertise in ketogenic therapy, involvement in the petition, and subsequent collaborations on metabolic initiatives, he was invited to join the manuscript (author C. P.)

## Methods

### Dataset

In 2025, following IRB determination that human subject’s approval was not required, we obtained the publicly available Change.org dataset from the petition creator (author C. P.). This study analyzed the data only in aggregate. No names, usernames or identifying information were reported, and no quotations are included in this manuscript, thereby minimizing the potential risk of re-identification. This petition and associated comments were publicly visible on the Change.org platform.

### Statistical analyses

Statistical analyses were performed using RStudio. We used top-down and bottom-up approaches to thematic analysis. For the top-down theme coding approach, four reviewers conducted iterative reviews of the comments to identify recurrent themes and quantify the number of comments associated with each theme. Differences in interpretation were resolved through group discussion until consensus was reached. Comments were assigned to themes based on consensus among the reviewers. Comments were able to be left uncharacterized and were able to be assigned to multiple themes.

Our bottom-up approach used Latent Dirichlet Allocation (LDA), a probabilistic topic modeling method (10), which models each comment as a mixture of latent topics defined by word distributions, which load together with varying degrees of strength. We used the Variational Expectation–Maximization (VEM) method.

Prior to modeling, text was pre-processed by removing stop words and nonwords (i.e., digits, URLs, emails, etc.), restricting analyses to English-language comments (only 3 comments were in other languages), and tokenizing unigrams (>4 characters, including word stems). Word stems were counted as the same word (e.g., “keto” and “ketogenic”). Unigrams were retained for topic modeling if they appeared ≥5 times overall. A document term matrix was generated to count the frequency of a given word per comment to input into the LDA model. Video comments and two off-topic comments were excluded from modeling, while all comments were retained for frequency analysis.

The number of topics was set to *K* = 6 based on human judgment of theme coherence (author S. M.). Although perplexity and coherence suggested a higher *K* (*K* = 29), the authors could not identify intuitive themes in these groupings. Therefore, many smaller *K*s were examined, with *K* = 6 yielding the most interpretable themes. We chose to keep the five strongest words per theme. Author S. M. interpreted the theme of title phrases based on the groups of words.

## Results

Between July 1, 2024, and December 6, 2025, the advocacy petition received 21,637 unique signatures and 1,105 comments from 5,914 cities across 151 countries. Table 1 shows signature counts and percentages for the top 10 most represented nations.

**Table 1:** Frequency (n) and proportion (%) of signatures in the top 10 most represented countries in descending order.

Top 10 most represented countries (descending)	Frequency and proportion of signatures N (%)
United States	15,598 (72%)
Canada	1,070 (5%)
United Kingdom	808 (4%)
Australia	639 (3%)
Philippines	268 (1%)
South Korea	222 (1%)
India	214 (1%)
Germany	200 (0.9%)
Netherlands	184 (0.9%)
New Zealand and South Africa (equal)	155 each (0.7% each)

**Figure 1:**
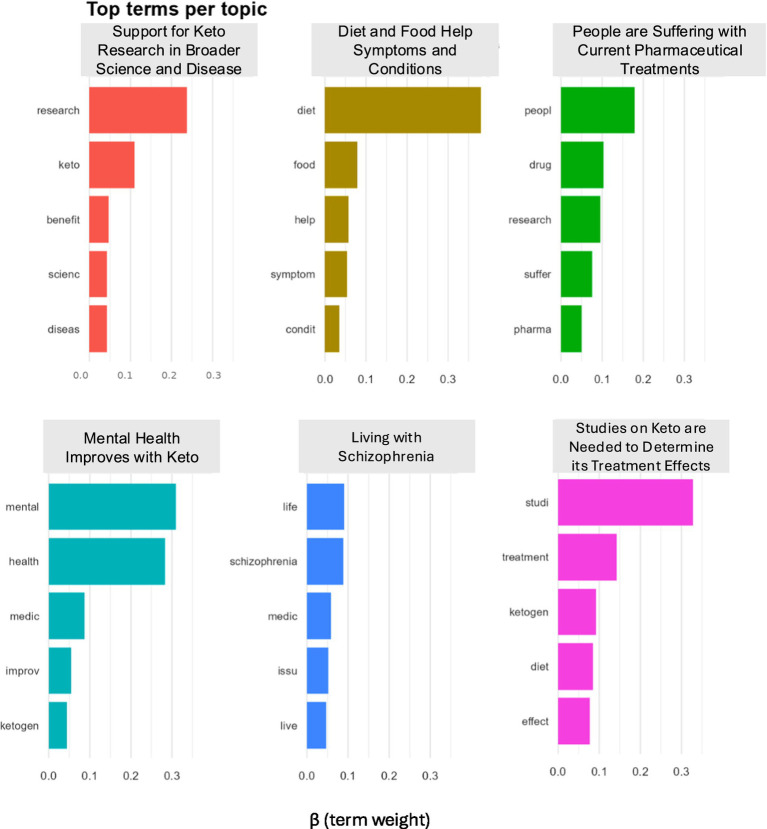
Topic modeling by LDA.

Table 2 shows the results of our theme coding and topic modeling of 1,105 petitioner comments. The top half of Table 2 displays the four themes identified through top-down theme coding. The bottom half of Table 2 lists the six themes derived from LDA topic modeling. Figure 1 shows LDA word groupings and term weights per topic.

**Table 2:** Reasons for petition signing (top-down theme coding and topic-modeling (LDA) methods).

Top-down theme coding	
Theme	N (%) *
Advocates for the research	560 (50.9%)
Ketogenic diet helped someone the commenter knew	187 (17.0%)
Ketogenic diet helped the commenter	148 (13.4%)
Administrative roadblocks and conflicts of interest	116 (10.5%)
Topic-modeling results (LDA)	
Theme	
Support for ketogenic diet research in broader science and disease	
Diet and food believed to help symptoms and conditions	
People are suffering with current pharmacologic treatments	
Mental health improves with ketogenic diet intervention	
Living with schizophrenia	
Studies are needed to determine [ketogenic diet] treatment effect	

*May not equal 100% due to uncharacterized comments and comments in more than one category.

## Discussion

This study reports quantitative and qualitative data from, to our knowledge, the largest petition examining respondents’ interest in dietary approaches to mental health and experiences related to the ketogenic diet. The petition, disseminated due to collective advocacy efforts via social media without incentives, collected over 21,000 signatures and more than 1,100 comments. Although largely U. S.-based, responses were from individuals in over 150 countries, indicating international engagement with the topic.

Petitioners’ comments revealed several themes. Top-down theme coding highlighted advocacy for research, reported success with the ketogenic diet for themselves or someone they knew, and concerns about study suspension. LDA topic modeling aided in identifying themes, including support for ketogenic research for broader science and disease; perceptions that diet helps manage symptoms; consensus that current pharmaceuticals are insufficient; testimonies of mental health improvement with ketogenic diets; the challenge of living with schizophrenia; and a strong call for research to determine the effectiveness of the ketogenic diet for mental illness.

Several important points emerge from these petition results. First, current pharmacologic treatments for schizophrenia do not resolve all symptoms, and many patients experience persistent side effects, and remission rates remain low (11). Nearly 40% are considered treatment-resistant after at least two antipsychotics (12). Many who signed the petition are calling for additional treatment options to be studied, including alternative approaches like dietary interventions.

Second, individuals with lived experience, family members, friends, and health care providers described perceived benefits associated with ketogenic diets and often characterized them as highly meaningful in the management of mental illness, including schizophrenia. Although these accounts do not provide evidence of efficacy, they illustrate that many petitioners reported positive experiences or observed improvements in mental health on the ketogenic diet among themselves or others, underscoring the importance of continued rigorous investigation of ketogenic dietary interventions.

Respondents also referred to the emerging literature on ketogenic interventions in schizophrenia. Although evidence remains preliminary and no adequately powered randomized controlled trials solely within a schizophrenia cohort have yet been published, existing studies include open-label clinical investigations (3, 4, 13), a retrospective analysis, (14) and case reports (5, 15–19). There are additional controlled studies of mixed diagnoses, including schizophrenia, currently undergoing analysis (20). These emerging data will need to be considered against potential contraindications and risks, side effects, and adherence challenges (21–23).

Lastly, many petitioners cited that they were advocates for expanding schizophrenia research. As schizophrenia has a high disease burden (24), and is often stigmatized, regulatory and administrative challenges are perceived as large barriers to conducting innovative clinical research in this population. Petitioners also expressed support for increased public and private investment in research to advance treatment development for schizophrenia. Private foundation funding and collaborations-for-care models have become important sources of support for schizophrenia research, particularly in the context of limited federal funding. For example, the number of NIMH-funded grants related to schizophrenia declined by 22% between 2016 and 2021, and at the time of this publication, there is only one active NIMH-funded pharmacologic clinical trial for schizophrenia in the U. S. (NCT05208190) (25).

Several limitations should be considered when interpreting these findings. Although the sample was large and geographically diverse, participation was influenced by both motivation and exposure. The petition was disseminated through social media and advocacy networks, including members of the international metabolic psychiatry community, nonprofit organizations, and mental health advocates. Specifically, following the discontinuation of the inpatient ketogenic diet trial in July 2024, the Change.org petition was initiated by an independent psychiatrist, Dr. Christopher Palmer, and shared with his approximately 350,000 followers on the following platforms: X (formerly Twitter), Facebook, Instagram, and LinkedIn. Additionally, Metabolic Mind and the Baszucki Foundation shared the petition information through the same channels, as well as YouTube, podcasts, and advocacy channels associated with the metabolic psychiatry community. Many social media health-and-wellness influencers with millions of followers, such as Andrew Huberman, signed and reposted the petition. Additional media attention, including opinion pieces and independent advocacy-oriented coverage, further increased the petition’s visibility and likely expanded its reach beyond local stakeholders. It is not possible to quantify the extent of sharing and advocacy, however the widespread engagement across over 150 countries suggests that the global metabolic community was active. Therefore, the results presented here represent a self-selected sample of individuals who chose to sign or comment on a publicly available petition supporting continued research into ketogenic dietary interventions for schizophrenia. Consequently, the signatures, comments, and geographic distribution reflect both participation, motivation, and the reach of the campaign, and should not be interpreted as measures of public opinion. It must also be pointed out that open online petitions can attract duplicate or incentivized signatures; however, Change. Org prevents duplicate signatures attached to the same email address.

Respondents were likely predisposed to support scientific research generally, and ketogenic diet research specifically. As such, the findings may not be representative of the broader population. In addition, only a subset of petitioners provided comments (5%), introducing a further level of self-selection. The comments analyzed do not capture perspectives related to skepticism of scientific merit, adherence challenges, or potential risks associated with ketogenic diet interventions, all of which are important considerations in discussions of implementation and clinical use.

Because respondents provided open-ended free-style text rather than answering prompts or structured survey questions, our analysis focused on identifying and characterizing themes rather than quantifying attitudes. Formal inter-rater reliability statistics were not calculated for the top-down thematic analysis, and topic prevalence estimates were not generated from the LDA model. Therefore, the findings should be interpreted as reflecting the range and nature of perspectives expressed rather than the relative frequency, prevalence, or importance of particular themes within the sample. Furthermore, the petition data are descriptive and provide no evidence regarding the efficacy or safety of ketogenic interventions.

In conclusion, this study highlights perspectives and experiences of a large, advocacy-driven, self-selected sample of petitioners interested in advancing research on novel approaches to schizophrenia treatment, including ketogenic dietary interventions. Responses frequently described perceived benefits associated with ketogenic diets and emphasized the importance of continued research in schizophrenia and serious mental illness. By analyzing comments from petitioners who voluntarily supported continued investigation in this area, we sought to document the experiences and perspectives of respondents rather than assess population-level attitudes or capture treatment efficacy. These findings underscore interest in further exploring the role of metabolic dysfunction in schizophrenia and support the need for rigorous evaluation of ketogenic dietary interventions through well-designed clinical studies.

## Data Availability

The original contributions presented in the study are included in the article/supplementary material, further inquiries can be directed to the corresponding author.

## Footnotes

1
https://www.change.org/p/help-us-protect-a-landmark-research-study-for-serious-mental-illness
